# The Red Boy, the Black Cat

**DOI:** 10.3201/eid2506.AC2506

**Published:** 2019-06

**Authors:** Byron Breedlove

**Affiliations:** Author affiliation: Centers for Disease Control and Prevention, Atlanta, Georgia, USA

**Keywords:** art science connection, emerging infectious diseases, art and medicine, zoonoses, about the cover, Francisco de Goya y Lucientes, Don Manuel Osorio Manrique de Zuniga, the Red Boy, the Black Cat, portrait, public health, animals

**Figure:**
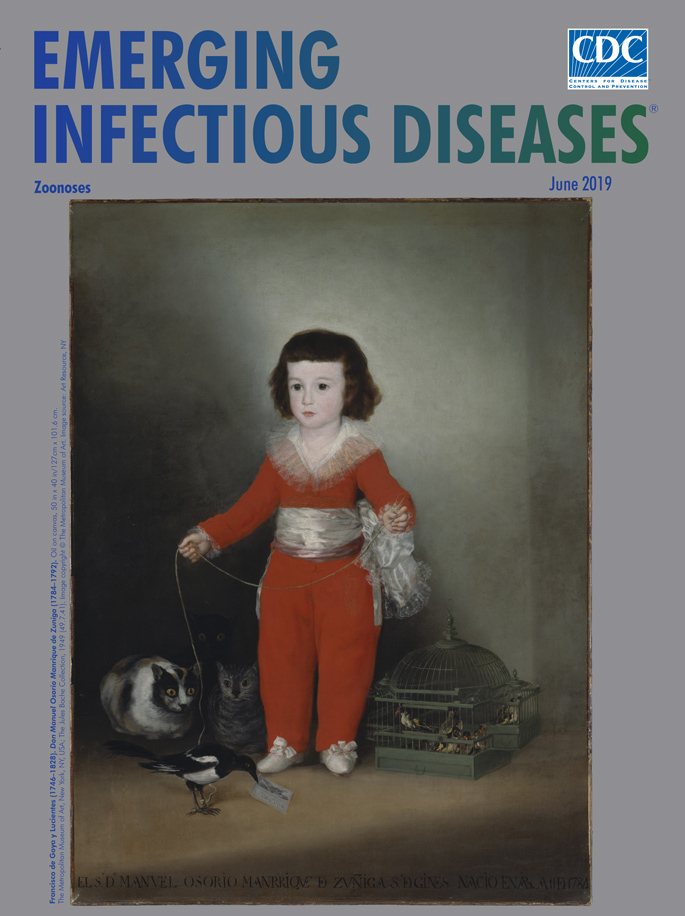
**Francisco de Goya y Lucientes (1746–1828). *Don Manuel Osorio Manrique de Zuniga* (1784–1792).** Oil on canvas, 50 in × 40 in/127 cm × 101.6 cm. The Metropolitan Museum of Art, New York, NY, USA; The Jules Bache Collection, 1949 (49.7.41). Image copyright © The Metropolitan Museum of Art. Image source: Art Resource, NY,

“Portraits of children accompanied by animals have a long tradition in Spanish painting,” notes the Metropolitan Museum of Art. Francisco de Goya contributed to that tradition with his “Portrait of Manuel Osorio Manrique de Zuniga.” But Goya also subverted tradition in this popular painting, commonly dubbed “The Red Boy,” by instilling a sense of discomfort and darkness to what could have been a simple proclamation of youthful innocence. 

Such tension appears in many of Goya’s portraits. Art critic and historian Laura Cumming notes “That Francisco de Goya . . . was a portrait painter first to last is a truth often neglected,” but she also observes a “strain of weirdness” in his portraits. Whether one agrees fully with her assessment, Goya’s art, which includes nearly 1,800 paintings, cartoons, murals, and etchings, defies convenient codification: he garners mentions as being among the last of the great masters and among the vanguard of modern artists.

Goya painted four portraits of members of the aristocratic Altamira family; of those, “The Red Boy” is the best known and most discussed. He completed those works nearly a decade before he suffered the debilitating, mysterious illness that caused headaches, vertigo, and delusions and is believed to have been triggered by his exposure to lead-based paints.

The Metropolitan Museum of Art Portraits offers this arm’s length description of this portrait: “Outfitted in a splendid red costume, the young boy, the son of the Count and Countess of Altamira, is shown with a pet magpie (which holds the painter’s calling card in its beak), a cage full of finches, and three wide-eyed cats. Although they add an engaging element for the viewer, Goya may have intended them as a reminder of the frail boundaries that separate the child’s world from the forces of evil, or as a commentary on the fleeting nature of innocence and youth. Manuel died at the tender age of eight.”

Manuel, resplendently dressed, dark eyes and hair, cuts a bold figure for one so young. He walks his magpie on a string while a trio of cats sizes up his pet, which grasps Goya’s calling card in its tilted beak. To the boy’s side, captive finches cluster near the bars of their green gazebo-like cage. Goya’s juxtaposition of cats and birds and of shadow and light creates a sense of latent unease and interjects an atmosphere of fidgeting discomfiture into the portrait. The black cat cloaked in shadows, nearly invisible save for its eyes, amplifies the undercurrent of menace. (This cat is so deep in the shadows that some critical assessments and many reproductions overlook it.)

Essayist and critic Morgan Meis observes that Goya portrays a child who, because of his social standing, is not allowed to dress or play like a child. Meis notes “The boy is trapped in layers of refinement that he cannot possibly understand,” and that “he knows nothing about court intrigues, just as he is unaware that the cats behind him are eagerly sizing up his pet bird.”

Cumming echoes those sentiments. She writes “the ‘Boy in Red’ may be a much-loved treasure, but the moppet in his pool of light is surrounded by more nightmare critters in the shape of owl-eyed cats waiting to savage a magpie, while sharp-beaked finches chatter in the darkness.”

Such elements of danger and tension in Goya’s work find traction with modern viewers. The trio of cats, especially the spectral shadow cat, seem intent on menacing the tethered magpie and caged birds. Both felids and fowl could themselves be potential sources of zoonotic diseases or harbor zoonotic disease vectors.

More than 60% of known infectious diseases in people are spread from animals, and 75% of new or emerging infectious diseases in people are spread from animals. Among those who may be at greater risk of acquiring zoonotic infections are young children who may not have learned how to practice effective hygiene or who have close contacts with pets or domestic animals. Human infections associated with cats include rabies, cat-scratch disease, capnocytophagosis, pasteurellosis, ringworm, sporothrichosis, tularemia, plague, Q fever, campylobacterosis, salmonellosis, *Escherichia coli* infections, cryptosporidiosis, giardiasis, and toxoplasmosis. Influenza, psittacosis, and salmonellosis are among the diseases birds may transmit to humans.

The cause of young Manuel’s death, if known, is not reported. Goya lived into his 80s and experienced a career that veered between triumph and torment. In the two centuries since Goya’s death, our knowledge about infectious diseases etiology and treatment has coalesced into an impressive body of knowledge. Still, like the nearly invisible black cat in Goya’s painting, some threats are harder than others to detect, and the need for constant vigilance remains a public health priority.
